# Continuous Sizing and Identification of Microplastics in Water

**DOI:** 10.3390/s23020781

**Published:** 2023-01-10

**Authors:** Felix Glöckler, Florian Foschum, Alwin Kienle

**Affiliations:** Institute for Lasertechnologies in Medicine and Metrology (ILM), Helmholtzstr. 12, 89081 Ulm, Germany

**Keywords:** microplastic, Monte Carlo simulations, Raman spectroscopy, light scattering

## Abstract

The pollution of the environment with microplastics in general, and in particular, the contamination of our drinking water and other food items, has increasingly become the focus of public attention in recent years. In order to better understand the entry pathways into the human food chain and thus prevent them if possible, a precise characterization of the particles concerning their size and material is indispensable. Particularly small plastic particles pose a special challenge since their material can only be determined by means of large experimental effort. In this work, we present a proof of principle experiment that allows the precise determination of the plastic type and the particle size in a single step. The experiment combines elastic light scattering (Mie scattering) with inelastic light scattering (Raman scattering), the latter being used to determine the plastic type. We conducted Monte Carlo simluations for the elastically scattered light for different kinds of plastics in a microfluidic cuvette which we could reproduce in the experiment. We were able to measure the Raman signals for different microplastics in the same measurement as the elastically scattered light and thereby determine their material. This information was used to select the appropriate Monte Carlo simulation data and to assign the correct particle size to different materials with only one calibration measurement.

## 1. Introduction

Despite the undeniably serious impact of microplastics on the environment [1,2], especially with regard to marine life [3,4,5], it is difficult to make a clear assessment of the extent to which humans are already affected. The best researched effects of plastics of all kinds are mainly of a physical nature, such as strangulation or the ingestion of larger quantities, which are, nevertheless, of no less importance [6]. However, small particles, i.e., particles smaller than 5 mm [7,8], account for the largest amount of plastic [6] unintentionally released into the environment. Although a connection between some diseases, e.g., fibrosis [9], and microplastic exposure has already been shown, a final assessment of the hazard potential of microplastics is difficult due to their rather low toxicity and large inertness. Particles smaller than 100 µm pose a particular challenge here. Not only do they account for a large part of the contamination of water resources used by civilization according to certain studies [10,11,12,13], but they are also particularly challenging in terms of their detection and correct identification.

A microplastic analysis is usually conducted by sampling and processing a certain volume of water [14] for an experiment. Many studies have been conducted in which water samples were sieved and filtered prior to the analysis of the particles contained therein. Here, only working under clean room conditions was shown to be an adequate experimental environment, because even in control samples a large number of fibers were found [15], which could be attributed to the contamination of the samples via airborne particles [16].

In the past, studies for the identification of plastic particles often used stereomicroscopic methods [17,18], but in recent times, it has been shown that these are extremely susceptible to a false positive assignment for microplastic fibers. For example, the differentiation of microplastics and sand does also not work reliably since it is only performed subjectively by the experimenter [18,19]. In addition, scanning electron microscopy [20] and X-ray spectroscopy [21] were used for the investigation, which allows a reliable differentiation from organic material and also provide information about the chemical composition and thus the plastic type but are only suitable for the investigation of a few individual particles due to the enormous experimental effort. For larger sample quantities, Raman microscopy [22,23] and Fourier transform infrared (FTIR) spectroscopy [24,25] have been proven to be reliable methods for the correct identification of microplastic particles. Likewise, more destructive thermoanalytical methods [26] as well as methods for direct staining [27] of the polymers have been successfully applied.

While FTIR spectroscopy is the most used spectroscopic method for the determination of microplastic materials [28], its application which yields the most stable spectra, the attenuated total reflection FTIR, still requires filtering and drying of the sample and is therefore susceptible to errors during the preparation process. However, Raman spectroscopy can readily be used on water samples directly [29,30]. Thus, avoiding many of the challenges which occur when measuring on dried filters. Apart from classic microscopic techniques on static samples, it is also possible to measure Raman spectra in a flow system, thus allowing the continuous measurement of particles without the need of removing a sample from the investigated liquid and therefore consequently avoiding the associated risks of contamination. For rather clean water with low biological load, such as tap water or process water in the food industry, Raman spectroscopy in flow systems can be a reliable method to monitor the microplastic load.

In this work, we present a setup that enables the detection and identification of microplastics. To determine the type of plastic, we use Raman spectroscopy in a flow system. We combine the knowledge about the particle material with our previous work [31], where we accurately sized particles with elastic light scattering and the a priori information about their refractive index. We show that material specific simulations allow the precise sizing of microplastic particles with only one calibration measurement.

## 2. Materials and Methods

### 2.1. Raman Scattering Detection

**Figure 1:**
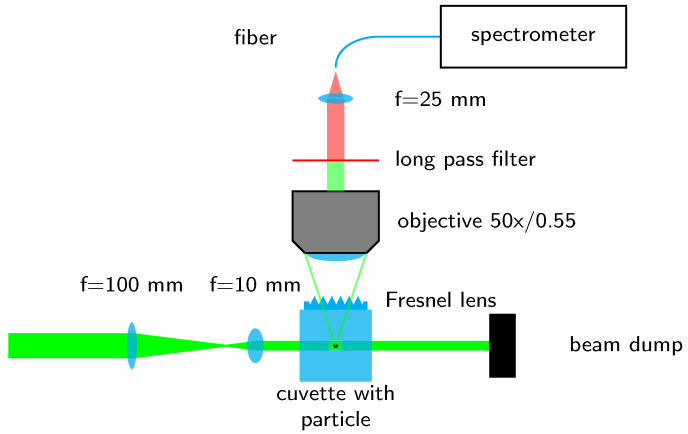
Arrangement to measure the Raman scattering.

**Figure 2:**
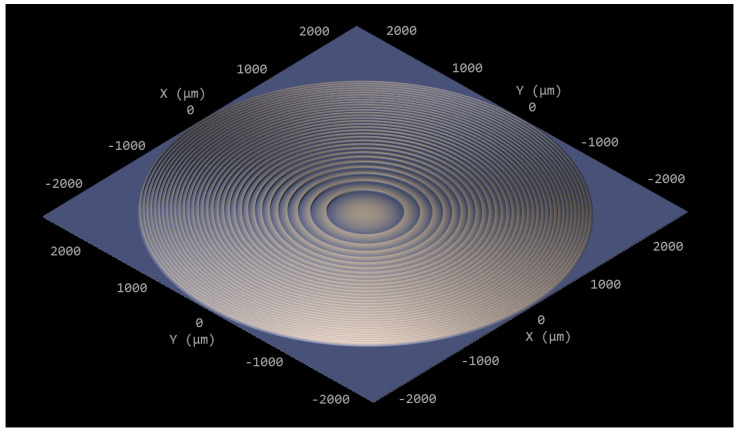
The printed Fresnel lens depicted with the preprint tool DeScribe.

The optomechanical setup, as shown in Figure 1, was built on a $600\times 800\;{\mathrm{mm}}^{2}$ baseplate, which was completely enclosed in a cardboard box for protection against stray light from the laser during operation. The laser that was used to excite the Raman scattering (RLTMLL-532-5W-5, Roithner Lasertechnik GmbH, Vienna, Austria) has a nominal power of 5.66 W at 532.163 nm with a line width of 88.5 pm. The laser operates in continuous-wave mode with a near ${\mathrm{TEM}}_{00}$ mode has a polarization ratio greater than 100:1 and a horizontal beam width ($1/e^{2}$) of 2.512 mm and a vertical height of 3.357 mm. Two lenses ($f_{1}=100\;\mathrm{mm}$ and $f_{2}=10\;\mathrm{mm}$) were used as a telescope to uniformly illuminate a quadratic fused silica cuvette with an outer dimension of 5 mm and a quadratic channel with a side length of 250 µm. After passing through the cuvette, the laser beam is then blocked by a beam stop. To collect the scattered light, a long-distance objective (LD-EC Epiplan-Neofluar 50×/0.55 HD DIC M27 air, Carl Zeiss AG, Oberkochen, Germany) with a numerical aperture (NA) of 0.55 was used perpendicularly to the illumination. The refraction at the fused silica–air boundary, which is a refraction away from the normal of the cuvette, effectively reduces the angular aperture of the used objective. Therefore, we designed a lens with a commercial optic design software (OpticStudio 22.2, Zemax LCC, Kirkland, WA, USA) to compensate the refraction at the boundary for the design wavelength of 630 nm. We chose the design wavelength of 630 nm due to the fact that when exciting plastics at 532 nm, the relevant Raman lines between 2800 ${\mathrm{cm}}^{-1}$ and 3200 ${\mathrm{cm}}^{-1}$ are located between 625 nm and 640 nm. The optical design assumed an object height of 100 µm and resulted in a plano-convex hyperbolic lens with a vertex radius of curvature of 3.010 mm and a conic constant of −1.292, which was realized as a Fresnel lens in order to save material and time during the manufacturing process. The resulting lens has a step height of 30 µm and an initial diameter of 2.151 mm which was enlarged by continuing the Fresnel pattern to 5 mm in order to cover the whole cuvette for easier positioning. For the manufacturing process, we used a two-photon polymerization printer (Photonic Professional GT+, Nanoscribe GmbH, Eggenstein-Leopoldshafen, Germany) and the commercially available resin IP-S (Nanoscribe GmbH, Eggenstein-Leopoldshafen, Germany). The lens was printed with a 5×5 mm base plate of 10 µm thickness to compensate eventual tilt of the printing substrate and for later handling. After the printing process, the lens was removed from the substrate and glued to the cuvette using a drop of IP-S, which was subsequently flood-cured. Figure 2 shows the model of the Fresnel lens on the base plate depicted with the preprint tool DeScribe (Nanoscribe GmbH, Eggenstein-Leopoldshafen, Germany), which is used to prepare models for printing. As printing parameters, we chose a (vertical) slicing and (horizontal) hatching distance of 0.3 µm and a writing speed of 100,000 µm/s.

The setup is completed by a long pass filter (cutoff 550 nm, FELH0550, Thorlabs, Newton, NJ, USA), which blocks the elastically scattered light. The inelastically scattered light, i.e., the Raman scattering, is then focused with a biconvex lens (f = 25 mm) into a fiber (NA = 0.22, core diameter = 1 mm) which is connected to a spectrometer (QE Pro-Raman-532-PLUS, Ocean Optics, Largo, FL, USA) with a 25 µm slit and a 1200 lines/mm grating which allows measurements up to 4500 ${\mathrm{cm}}^{-1}$ Raman shift.

### 2.2. Elastic Scattering Detection

While the inelastically scattered light, i.e., the Raman scattering, is used for the determination of the material type, the elastic scattering is used to determine the size of the particle. While the type of material is determined via a spectrum, the form of which does ideally not change with the position of the particle inside the cuvette, the size is connected to the height of the elastic scattering signal, which can change with the particle’s position inside the cuvette. To investigate the influence of the position inside the cuvette on the signal strength, we conducted GPU–accelerated Monte Carlo (MC) simulations following the principles of Alerstam et al. [32,33]. We investigated 9 different positions inside the cuvette, one being the center, while the other 8 were located 75 µm away from the center in one or two lateral directions. These positions were chosen according to the results of Segré and Silberberg [34,35] that a rigid sphere that is transported in a Poiseuille flow reaches an equilibrium position at ≈0.6·r from the axis of a tube, where *r* is the tube radius. The simulation was performed by placing a point source, which emitted “photons” according to a scattering phase function calculated with the Mie theory [36,37], inside the cuvette on the respective positions. The detectors were modeled as fiber detectors with a diameter of 1 mm and NA = 0.22. The output of the simulation is the number of detected “photons” divided by the total number of “photons” and the sensor area. This number is then multiplied with the scattering cross section of the simulated particle to factor in the varying magnitude of the scattering itself, which yields a dimensionless number that can be correlated to the detected intensity. We simulated spheres with a diameter from 1 µm up to 199 µm in 2 µm steps at the 9 positions. For the simulations that are described in the following paragraph, we considered the particles to be polystyrene spheres. We note that larger particles can no longer occupy these positions and are located closer to the center of the cuvette.

**Figure 3:**
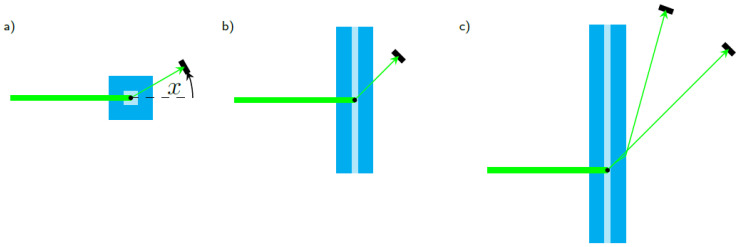
(**a**) Sensor rotated radially and (**b**) vertically and also with (**c**) consideration of the refraction at the interfaces. All given angles are with respect to the direction of illumination.

Due to the quadratic shape of the cuvette, it is evident that some positions for the detector sensor are less favorable than others. In the search for a suitable sensor position we considered three different geometries that are depicted in Figure 3. The most obvious case is shown in Figure 3a where the sensors are located in the plane perpendicular to the axis of the channel. The channel of the cuvette is the axis of rotation and the angle *x* in respect to the direction of the illumination.

**Figure 4:**
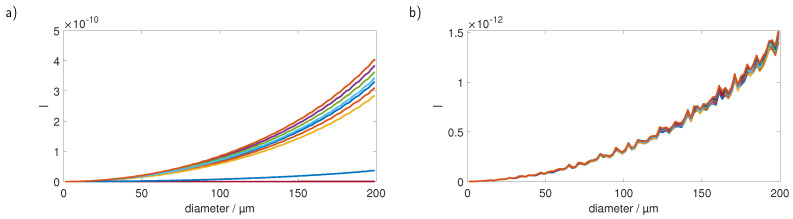
MC simulation of a fiber detector positioned in 5 mm distance to the cuvette at (**a**) $30^{\circ }$ and (**b**) $90^{\circ }$. Each color corresponds to a different particle position.

Placing a sensor at $x=45^{\circ }$ in said plane would obviously result in adverse effects on the detected signal due to the effects of the corner of the cuvette on the light propagation. Figure 4 shows a simulation where sensors were placed at 5 mm distance to the cuvette at different angles. While the signals for a sensor positioned at $30^{\circ }$, as shown in Figure 4a, exhibit a strong dependence of the signal strength on the particle’s position, they are also rather smooth and do not show pronounced Mie oscillations. Figure 4b shows results for a sensor position at $x=90^{\circ }$. Although the signal does not vary much between the positions, it shows strong Mie oscillations in its course, making this sensor position also rather unsuitable for precise particle sizing.

**Figure 5:**
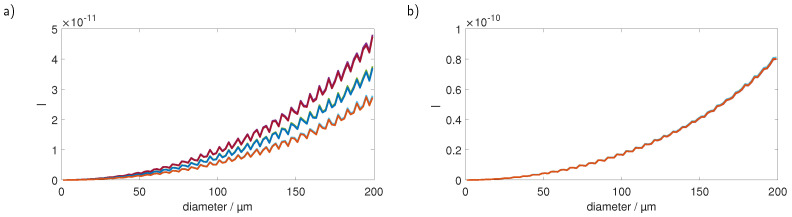
MC simulation of fiber detectors at (**a**) $45^{\circ }$ and (**b**) ≈70${}^{\circ }$. The latter angle is obtained due to the refraction of the light that was originally originating at $45^{\circ }$ from the scatterer. Each color represents a different particle position.

Due to the less prominent Mie oscillations and the larger scattering signals, we focused on finding a suitable sensor position in forward scattering direction. Therefore, we investigated detector positions in a plane spanned by the illumination and the channel axis of the cuvette. In a further step, we also considered the refraction of the scattered light at the surface boundaries. Figure 5a shows the detected intensities of a sensor detecting light at $45^{\circ }$ as depicted in Figure 3b. While the signal shows a strong dependence on the particle position as well as prominent Mie oscillations, it also shows that, due to the symmetry of the detection arrangement, 3 of the 9 positions each produce an almost identical signal. Figure 5b shows the results for a sensor that is rotated in such a way that the refraction at the different boundaries (water–fused silica, fused silica–air) is taken into account, which is sketched in Figure 3c. The light that is originally scattered at an angle of $45^{\circ }$ leaves the cuvette with ≈70${}^{\circ }$. Rotating the sensor toward this angle and positioning it accordingly yields a signal that is not only smooth in terms of the Mie oscillations but also shows almost no dependency on the position inside the cuvette. This effect was observed for several angles. However, for the scattering at originally $45^{\circ }$, the effects of the particle position were the smallest and all later measurements were performed this way.

## 3. Results

### 3.1. Raman Signals

**Figure 6:**
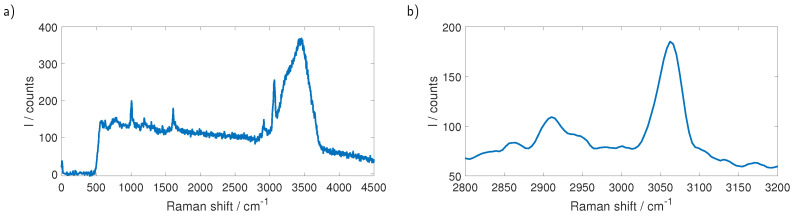
(**a**) Raman spectrum of a single PS sphere with 101 µm diameter. The relevant lines for the differentiation of different types of plastic are between 2800 cm${}^{-1}$ and 3200 cm${}^{-1}$ for 532 nm excitation and partly hidden in the broad Raman response of water. (**b**) The same measurement after subtracting the background and smoothing the data with a Savitzky–Golay filter. The relevant Raman lines of PS at 2855 cm${}^{-1}$, 2907 cm${}^{-1}$ and 3058 cm${}^{-1}$ are visible.

Measurements were performed by preparing suspensions of microbeads (microParticles GmbH, Berlin, Germany) with purified water (Ampuwa, Fresenius Kabi, Bad Homburg, Germany). We used polystyrene (PS) and polymethyl methacrylate (PMMA) particles and the sizes of the beads ranged from 4.21 µm to 101 µm in the case of PS while for PMMA we examined beads with 101 µm diameter. The micro-fluidic system was driven by a programmable peristaltic pump (iPump 2F, Landgraf Laborsysteme HLL GmbH, Langenhagen, Germany) with a flow of 30 mL/h. The full Raman spectrum of a single PS particle with a diameter of 101 µm is shown in Figure 6a. Distinguishing between different types of plastic while exciting with 532 nm can be done by looking at the Raman lines between 2800 cm${}^{-1}$ and 3200 cm${}^{-1}$. Although these lines are overlapping with the broad water peak, belonging to the OH-stretching, between 3000 cm${}^{-1}$ and 3600 cm${}^{-1}$, they are plainly visible. Subtracting the background, which was obtained by measuring pure water without particles, and smoothing the data using a Savitzky–Golay filter yields the data shown in Figure 6b, which shows the Raman lines at 2855 cm${}^{-1}$, 2907 cm${}^{-1}$ and 3058 cm${}^{-1}$ that are expected to be seen for PS. Using the described method, we were able to measure the Raman spectra of PS spheres down to 26.15 µm diameter.

**Figure 7:**
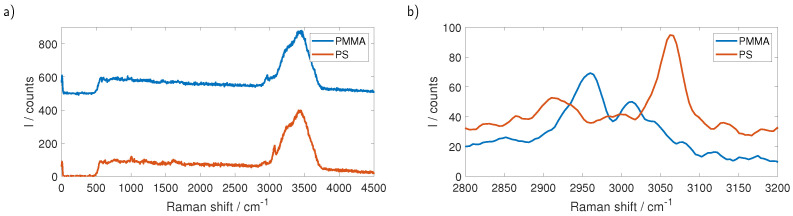
(**a**) Measurement of a sample containing PS as well as PMMA. For better visibility, the measurements are stacked by adding an offset. Although both plastics have their relevant lines in the area of the water response, they are already distinguishable in the raw data. (**b**) The same measurement after subtracting the background and smoothing the data with a Savitzky–Golay filter without the offset. The spectra are easily distinguished.

Figure 7 shows the measurement on a sample that contains PMMA as well as PS particles. The two types can be told apart in the raw data, shown in Figure 7a, and smoothing the data and subtracting the background, as shown in Figure 7b, further clarifies the differences.

### 3.2. Size Determination

**Figure 8:**
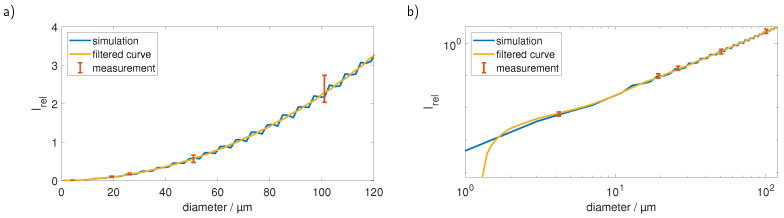
The simulated curve depicted together with the Savitzky–Golay smoothed one and the measurements in a (**a**) linear and a (**b**) double-logarithmic plot. Scaling the simulation to the measurement was done by dividing every point of the simulated curve through the simulated value at 50.7 µm and then multiplying it with the measured intensity for the sphere with a diameter of 50.7 µm.

To determine the size of the particles we used the detection geometry, described in Section 2.2, by bringing a bare fiber with the aforementioned angle close to the cuvette. As in the simulation presumed, the fiber had a diameter of 1 mm and NA = 0.22 and was connected to an amplified photodiode (OE-300-SI-10-FST, FEMTO Messtechnik GmbH, Berlin, Germany) that was operated in alternating current mode and connected to a digital oscilloscope (PicoScope 5444D, Pico Technology, Saint Neots, UK) to monitor the signals. For the unambiguous determination of the size of a particle, we smoothed the MC simulation data with a Savitzky–Golay filter to get a monotonous ascending curve. We performed a calibration measurement with monodisperse PS spheres with a diameter of 50.7 µm diameter and scaled the simulated curve according to this measurement. We then measured particles with different sizes (4.21 µm, 19.3 µm, 26.15 µm and 101 µm) and calculated their sizes with the scaled calibration curve by interpolating the measured signal between the nearest neighbours on the curve. Both the scaled initial simulation and the Savitzky–Golay filtered curve, as well as the measurements with the standard deviation as the error bar, are shown in Figure 8a in linear and in Figure 8b in double-logarithmic depiction. We assumed the voltage measured by the diode to be linear to the intensity of the scattered light.

**Figure 9:**
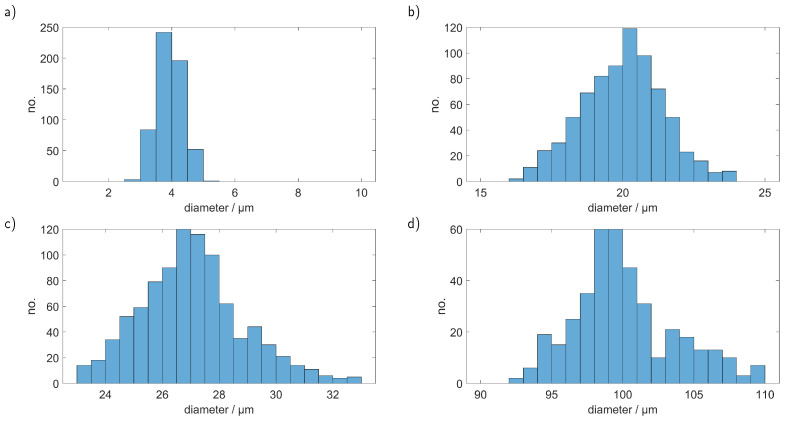
Determined sizes of PS particles with a diameter of (**a**) 4.21 µm, (**b**) 19.3 µm, (**c**) 26.15 µm and (**d**) 101 µm.

Figure 8 clearly shows that we could reproduce the conducted simulations and therefore achieve a correct sizing of the particles. However, the smoothing of the simulated curve, which is necessary for an unambiguous assignment of the signals, introduces first errors. Furthermore, at first glance, it seems that the precision decreases with increasing particle size, a look at the double-logarithmic depiction and also the retrieved particle sizes, which are depicted in Figure 9, shows that the relative error remains in the same order of magnitude and a reasonable precise sizing is possible over the observed range.

The observed deviations from the value given by the manufacturer can also be partially attributed to the smoothing of the curve. The measurement of the 19.30 µm particles, for example, lies almost exactly on the value of the simulation, but at this point, an oscillation was smoothed, which is why the size is slightly overestimated. In the measurement of the 101 µm particles, where the central size was determined precisely, an oscillation would follow that was smoothed so that higher signals are assigned a larger size by the slower rising curve.

**Figure 10:**
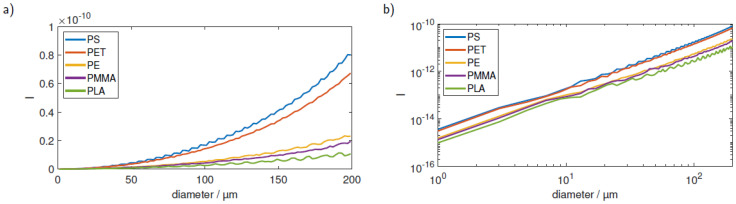
Calibration for five typical microplastic materials in (**a**) linear and (**b**) double logarithmic plots. The signal course is comparable between all materials; however, the absolute values differ largely.

Apart from the size, the intensity of the scattered light is also heavily dependent on the refractive index of the investigated plastic particles. Figure 10 shows the curves for five commonly found plastic materials (PS, polyethylene terephthalate (PET), polyethylene (PE), PMMA, polylactic acid (PLA)) in water in linear (a) and double logarithmic (b) plots. It is evident that for particles, such as PMMA, with a lower refractive index the sole comparison of the signal strength with the PS calibration curve yields wrong results.

**Figure 11:**
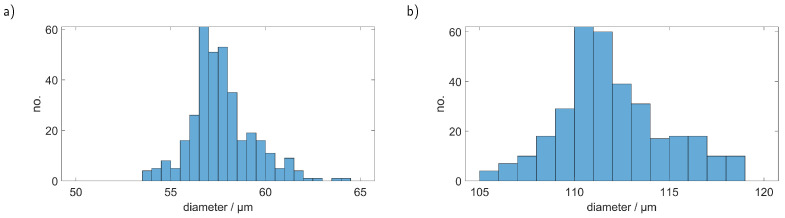
Determined sizes of PMMA particles with a diameter of 101 µm with (**a**) the original PS calibration curve and (**b**) with the PMMA curve that was corrected according to the simulation and the single calibration point with the 50.7 µm PS sphere.

Figure 11a shows the sizes we obtained from measured PMMA spheres with 101 µm diameter using the original PS calibration curve. It is quite obvious that PMMA particles, with their lower refractive index mismatch to water, are scattering the light much weaker in comparison to PS. Thus, the same particle sizes yield different scattering signals leading to the wrong assigned diameter as shown in Figure 11a. Using the knowledge about the material type of the investigated particles (and therefore their refractive index), which can be obtained from the Raman measurement we can, while still using only one PS calibration measurement, correct the determined sizes. Figure 11b shows the sizes that were obtained after smoothing the PMMA simulation curve with a Savitzky–Golay filter and scaling it accordingly to the PS calibration measurement.

While it was not possible to reach the same precision as in the PS measurements, we still managed to reduce the error for the size detection from over 40 % to around 10 %.

## 4. Conclusions

In this work, we presented a setup that served as a proof of principle that it is possible to detect and characterize microplastic particles using both elastic and inelastic light scattering. We achieved a precise sizing of the particles that had the same material as the calibration particle and managed to improve the determined sizes of particles made by another material using the knowledge of its refractive index which can be obtained by identifying them utilizing Raman measurements. The sizing is accurate enough to serve as a vital point of information in assessing the risks of ingestion. Placing additional sensors at different angles could also give further information about the shape of the particle. We plan to investigate this using custom microplastic particles printed with a two-photon polymerization process.

Concerning the measurement speed, the Raman measurements were the limiting factor in this experiment since the particles remained only for ≈3.5 ms in the laser beam at a 30 mL/h flow rate. With this, we could measure Raman signals down to a particle size of 26.15 µm and clearly distinguish between PS and PMMA particles. We think our approach to size and characterize microplastics is vastly promising since there is still room to improve the experiment, such as using an objective with higher NA or a larger slit at the spectrometer and limiting the detection only to the relevant lines between 2800 cm${}^{-1}$ and 3200 cm${}^{-1}$. The largest effect on increasing the flow rate would be achieved by using a larger cuvette. The greater distances between the particles would mostly affect the determination of the size and not the material due to the different positions in the cuvette. However, this problem can be overcome by increasing the sensor distances, which makes the path length differences small again in relation. Additional optics in front of the sensors can also increase the detected solid angle and thus dampen the Mie oscillations.

## Data Availability

No new data were created or analyzed in this study. Data sharing is not applicable to this article.

## References

[1] Rhodes C.J. (2018). Plastic pollution and potential solutions. Sci. Prog..

[2] Ritchie H., Roser M. (2018). Plastic pollution. Our World Data.

[3] Haward M. (2018). Plastic pollution of the world’s seas and oceans as a contemporary challenge in ocean governance. Nat. Commun..

[4] Thushari G.G.N., Senevirathna J.D.M. (2020). Plastic pollution in the marine environment. Heliyon.

[5] Wilcox C., Van Sebille E., Hardesty B.D. (2015). Threat of plastic pollution to seabirds is global, pervasive, and increasing. Proc. Natl. Acad. Sci. USA.

[6] Bertling J., Bertling R., Hamann L. (2018). Kunststoffe in der Umwelt: Mikro- und Makroplastik.

[7] Thompson R.C., Olsen Y., Mitchell R.P., Davis A., Rowland S.J., John A.W., McGonigle D., Russell A.E. (2004). Lost at sea: Where is all the plastic?. Science.

[8] Wright S.L., Thompson R.C., Galloway T.S. (2013). The physical impacts of microplastics on marine organisms: A review. Environ. Pollut..

[9] An R., Wang X., Yang L., Zhang J., Wang N., Xu F., Hou Y., Zhang H., Zhang L. (2021). Polystyrene microplastics cause granulosa cells apoptosis and fibrosis in ovary through oxidative stress in rats. Toxicology.

[10] Oßmann B.E., Sarau G., Holtmannspötter H., Pischetsrieder M., Christiansen S.H., Dicke W. (2018). Small-sized microplastics and pigmented particles in bottled mineral water. Water Res..

[11] Erni-Cassola G., Gibson M.I., Thompson R.C., Christie-Oleza J.A. (2017). Lost, but found with Nile red: A novel method for detecting and quantifying small microplastics (1 mm to 20 μm) in environmental samples. Environ. Sci. Technol..

[12] Schymanski D., Goldbeck C., Humpf H.U., Fürst P. (2018). Analysis of microplastics in water by micro-Raman spectroscopy: Release of plastic particles from different packaging into mineral water. Water Res..

[13] Koelmans A.A., Nor N.H.M., Hermsen E., Kooi M., Mintenig S.M., De France J. (2019). Microplastics in freshwaters and drinking water: Critical review and assessment of data quality. Water Res..

[14] Ivleva N.P., Wiesheu A.C., Niessner R. (2017). Microplastic in aquatic ecosystems. Angew. Chem. Int. Ed..

[15] Mintenig S., Löder M., Primpke S., Gerdts G. (2019). Low numbers of microplastics detected in drinking water from ground water sources. Sci. Total Environ..

[16] Wesch C., Elert A.M., Wörner M., Braun U., Klein R., Paulus M. (2017). Assuring quality in microplastic monitoring: About the value of clean-air devices as essentials for verified data. Sci. Rep..

[17] Li J., Liu H., Chen J.P. (2018). Microplastics in freshwater systems: A review on occurrence, environmental effects, and methods for microplastics detection. Water Res..

[18] Shim W.J., Hong S.H., Eo S.E. (2017). Identification methods in microplastic analysis: A review. Anal. Methods.

[19] Song Y.K., Hong S.H., Jang M., Han G.M., Rani M., Lee J., Shim W.J. (2015). A comparison of microscopic and spectroscopic identification methods for analysis of microplastics in environmental samples. Mar. Pollut. Bull..

[20] Zbyszewski M., Corcoran P.L., Hockin A. (2014). Comparison of the distribution and degradation of plastic debris along shorelines of the Great Lakes, North America. J. Great Lakes Res..

[21] Vianello A., Boldrin A., Guerriero P., Moschino V., Rella R., Sturaro A., Da Ros L. (2013). Microplastic particles in sediments of Lagoon of Venice, Italy: First observations on occurrence, spatial patterns and identification. Estuar. Coast. Shelf Sci..

[22] Araujo C.F., Nolasco M.M., Ribeiro A.M., Ribeiro-Claro P.J. (2018). Identification of microplastics using Raman spectroscopy: Latest developments and future prospects. Water Res..

[23] Käppler A., Fischer D., Oberbeckmann S., Schernewski G., Labrenz M., Eichhorn K.J., Voit B. (2016). Analysis of environmental microplastics by vibrational microspectroscopy: FTIR, Raman or both?. Anal. Bioanal. Chem..

[24] Tagg A.S., Sapp M., Harrison J.P., Ojeda J.J. (2015). Identification and quantification of microplastics in wastewater using focal plane array-based reflectance micro-FT-IR imaging. Anal. Chem..

[25] Cabernard L., Roscher L., Lorenz C., Gerdts G., Primpke S. (2018). Comparison of Raman and Fourier transform infrared spectroscopy for the quantification of microplastics in the aquatic environment. Environ. Sci. Technol..

[26] Dümichen E., Eisentraut P., Bannick C.G., Barthel A.K., Senz R., Braun U. (2017). Fast identification of microplastics in complex environmental samples by a thermal degradation method. Chemosphere.

[27] Maes T., Jessop R., Wellner N., Haupt K., Mayes A.G. (2017). A rapid-screening approach to detect and quantify microplastics based on fluorescent tagging with Nile Red. Sci. Rep..

[28] Hanvey J.S., Lewis P.J., Lavers J.L., Crosbie N.D., Pozo K., Clarke B.O. (2017). A review of analytical techniques for quantifying microplastics in sediments. Anal. Methods.

[29] Kniggendorf A.K., Meinhardt-Wollweber M. (2011). Of microparticles and bacteria identification–(resonance) Raman micro-spectroscopy as a tool for biofilm analysis. Water Res..

[30] Kniggendorf A.K., Wetzel C., Roth B. (2019). Microplastics detection in streaming tap water with Raman spectroscopy. Sensors.

[31] Müller D., Glöckler F., Kienle A. (2019). Application of Mie theory for enhanced size determination of microparticles using optical particle counters. Appl. Opt..

[32] Alerstam E., Lo W.C.Y., Han T.D., Rose J., Andersson-Engels S., Lilge L. (2010). Next-generation acceleration and code optimization for light transport in turbid media using GPUs. Biomed. Opt. Express.

[33] Alerstam E., Svensson T., Andersson-Engels S. (2009). User Manual and Implementation Notes.

[34] Segre G., Silberberg A. (1962). Behaviour of macroscopic rigid spheres in Poiseuille flow Part 1. Determination of local concentration by statistical analysis of particle passages through crossed light beams. J. Fluid Mech..

[35] Segre G., Silberberg A. (1962). Behaviour of macroscopic rigid spheres in Poiseuille flow Part 2. Experimental results and interpretation. J. Fluid Mech..

[36] Bohren C.F., Huffman D.R. (2008). Absorption and Scattering of Light by Small Particles.

[37] Mie G. (1908). Beiträge zur Optik trüber Medien, speziell kolloidaler Metallösungen. Ann. Phys..

